# Experiences of parents and adolescents throughout the solid-organ transplantation process: a focus group study

**DOI:** 10.3389/fpsyt.2026.1633641

**Published:** 2026-08-05

**Authors:** Jessica Garrido-Bolton, Rocío de la Vega, María Fe Bravo-Ortiz, Eduardo Fernández-Jiménez

**Affiliations:** 1 Department of Child and Adolescent Psychiatry, Clinical Psychology and Mental Health, La Paz University Hospital, Madrid, Spain; 2 Hospital La Paz Institute for Health Research (IdiPAZ), Madrid, Spain; 3 Department of Personality, Evaluation and Psychological Treatment, University of Málaga, Málaga, Spain; 4 Instituto Biomédico de Málaga – IBIMA Plataforma Bionand, Málaga, Spain; 5 Department of Psychiatry, Clinical Psychology and Mental Health, La Paz University Hospital, Madrid, Spain; 6 Department of Psychiatry, Universidad Autónoma de Madrid (UAM), Madrid, Spain; 7 Centro de Investigación Biomédica en Red de Salud Mental (CIBERSAM), Instituto de Salud Carlos III, Madrid, Spain; 8 Faculty of Law, Education and Humanities, Universidad Europea de Madrid, Madrid, Spain

**Keywords:** biopsychosocial model, focus groups, solid-organ transplantation, thematic framework analysis, transplanted adolescents and parents, unmet care needs

## Abstract

**Background:**

Solid-organ transplantation is the optimal treatment for children and adolescents with end-stage organ failure. However, the biopsychosocial challenges faced jointly by this paediatric population have received little attention. This study is one of the few qualitative studies based on focus groups to identify the unmet care needs perceived by transplanted adolescents and parents of paediatric transplant recipients throughout the pre-, peri-, and post-surgical phases of various types of solid-organ transplantation.

**Methods:**

Qualitative data was collected using a purposive sample selected according to the concept of information power at the Children’s Hospital of La Paz University Hospital (Madrid, Spain). The adolescent dataset comprised one focus group with four adolescent transplant recipients and one additional participant who completed an individual in-depth interview using the same interview guide. The parent dataset consisted of one focus group with five parents of paediatric transplant recipients. A biopsychosocial theoretical framework and semi-structured moderating guide were used. Participants first answered open-ended questions about desired healthcare improvements, followed by targeted questions on biomedical, psychological, and social issues that were not spontaneously addressed. No further focus groups with additional participants were required, as theoretical sufficiency had been attained. Thematic framework analysis was used to examine the data, involving familiarisation, identifying a thematic framework, indexing, charting, mapping, and interpretation. Finally, the COnsolidated criteria for REporting Qualitative research (COREQ) were followed.

**Results:**

Significant unmet care needs were identified throughout the transplantation process, particularly in the social and psychological domains. Participants reported concerns regarding long travel distances to hospitals, extended waiting times for outpatient visits, limited access to academic support, and insufficient peer and family support. The need for a designated social work contact person, compassionate healthcare interactions, and consistent access to mental health care was also highlighted.

**Conclusion:**

The findings will guide the development of prehabilitation programs and longitudinal support interventions for solid-organ transplant patients and their families, emphasising the need to understand their experiences and unmet needs to enhance healthcare and clinical outcomes.

**Clinical Trial Registration:**

The study protocol was registered at clinicaltrials.gov (NCT05441436). De-identified individual participant data will be made available in the **Supplementary Material File**.

## Introduction

Solid-organ transplantation is the best treatment option for most children and adolescents with end-stage organ failure (1). Despite advancements in allograft survival rates in paediatric populations following solid-organ transplant, to date, understanding the challenges faced by survivors, such as the impact on biopsychosocial outcomes, has received little attention (2).

Children with end-stage organ failure inherently face life-threatening conditions and potentially traumatic experiences, including major surgeries, intensive care unit (ICU) admissions, frequent invasive procedures, and ongoing hospital visits. These medical challenges are compounded by limited social interactions, school absences, and restricted participation in typical childhood activities, collectively exerting a substantial negative impact on the quality of life of both patients and their families (3).

Mental Health Consultation-Liaison teams serve essential functions within transplant clinical settings, including evaluating living donor candidates, assessing recipients’ comprehension of the transplantation process, monitoring mental health status, functioning and quality of life, addressing potential psychopathological comorbidities, and providing psychological support to both patients and their families (4).

Previous research has provided valuable insights into parental stress, family functioning, and coping strategies in paediatric transplant populations, as well as in families of children with chronic illnesses (5–9). However, most studies have focused exclusively on parents, specific post-transplant periods, or particular contextual stressors such as the COVID-19 pandemic. While quantitative research has significantly advanced the clinical management of paediatric transplant recipients, qualitative studies remain scarce and are typically limited to specific organ types.

To address this gap, the present study investigated the subjective experiences of adolescent solid-organ transplant recipients and parents of paediatric transplant recipients across the transplantation process. To the best of our knowledge, this is among the few qualitative studies using focus groups to explore unmet care needs in this population across the pre-, peri-, and post-transplant phases, involving multiple organ transplant types. In this study, unmet care needs are defined as biomedical, psychological, and social needs that patients and parents perceived as not being adequately identified, addressed, or supported during the transplantation journey. This qualitative approach contributes to the humanisation of scientific and care activities by incorporating the perspectives, preferences, values, and expectations of patients and families. Consistent with a family-centred care model (10), it promotes a comprehensive and holistic approach in which patients and their parents are actively represented and considered.

The Children’s Hospital of La Paz University Hospital in Madrid is a national referral centre for paediatric healthcare in Spain. It is recognised for performing all major types of paediatric solid-organ transplantation and for managing other complex paediatric conditions. According to the 2022 annual report of the National Transplant Organization, La Paz was among the leading centres in Spain for paediatric transplants, making it a key site for clinically relevant paediatric data (11).

Accordingly, this study aimed to explore the perceived unmet care needs of adolescent solid-organ transplant recipients and parents of paediatric transplant recipients across the biomedical, psychological, and social domains throughout the transplantation process. We expect that these findings will provide essential novel information for the design of future prehabilitation and longitudinal support interventions for paediatric transplant recipients (i.e. to prepare the patient to be in the best possible state before and after surgery).

## Methods

### Study design

The focus group method was selected because of its group dynamics, which can generate rich data, particularly in situations where prior knowledge is limited (12). Focus groups and semi-structured interviews are widely recognised as the primary methods used in qualitative research (13). Non-quantitative methods have been increasingly used by paediatric psychologists and other researchers to enhance scientific understanding and offer novel insights into healthcare (14). These methods examine how children and their families experience and respond to health-related challenges, allowing researchers to better understand the phenomena under study (15).

To ensure the accuracy and transparency of the reporting of this qualitative study, we closely followed the COnsolidated criteria for REporting Qualitative research (COREQ). This involved a 32-item checklist developed specifically for reporting research related to interviews and focus groups (13).

### Sample

Participants were recruited by the Mental Health Consultation-Liaison team of the Children’s Hospital of La Paz University Hospital (Madrid, Spain) using purposively selected sampling to maximise variation in transplant types and participant perspectives, while ensuring “information power” (16). The adolescent group included individuals who were aged 12–17 years at the time of transplantation, who had undergone solid-organ transplantation 6 months to 2 years prior to the study, were fluent in Spanish, and were first-time transplant recipients. Adolescents were deliberately selected to represent different types of solid-organ transplants and to capture a range of experiences across the pre-, peri-, and post-transplant trajectories.

A separate group of parents was included to provide complementary perspectives on caregiving and family-level challenges. These parents were not necessarily the parents of the adolescent participants to potentially broaden the number of experiences. The group comprised parents of paediatric transplant recipients who were aged 7–13 years at the time of transplantation. Although the study focused on adolescent experiences, including parents of younger recipients broadened the range of family experiences and reflected recruitment constraints due to limited availability of eligible parents within the adolescent subgroup.

Again, the selection criteria for parents prioritised diversity in transplant experiences, including a parent who had been a donor and an immigrant family facing additional challenges. One exception was made for a mother and her adolescent child who received a lung transplant. The limited number of participants who had undergone transplantation for this organ type required their joint participation in this study. Participants in both groups were selected with the prior understanding that they would actively engage in focus group discussions.

To ensure information power, more than one participant from the most common transplant types was included—kidney transplants in the adolescent group and heart transplants in the parent group—allowing the study to reflect typical experiences while capturing the breadth of challenges across the target population.

One adolescent was unable to attend the scheduled focus group and was subsequently invited to participate in an individual in-depth interview following the same discussion guide and conditions as the focus group. Consequently, the adolescent data consisted of a focus group with four participants and one individual interview, while the parent focus group included five participants. The total sample comprised 10 participants (*n* = 10).

### Procedure

The parents of each selected participant or the patients themselves, when of legal age, were contacted by a research team member via telephone to confirm eligibility and provide details about the focus group procedure. All participants, including minors, were fully informed of the nature and purpose of the study prior to participation. All contacted participants wished to participate in the study (minors provided assent, indicating their voluntary willingness to participate). Parents of patients (or, when applicable, patients of legal age) who provided written informed consent were scheduled to participate.

Two group sessions were conducted (one session per participant type: patients and parents), lasting approximately 90 min each, until theoretical sufficiency was attained (according to theme recurrence, when no new information of value was obtained). Focus groups were conducted online to facilitate participation by adolescents and parents across different regions of Spain without the need for long-distance travel. The individual in-depth interview was also conducted online.

The interview guide employed in both focus groups, was based on the biopsychosocial theoretical framework. The study questions were developed by researchers and clinicians experienced in paediatric transplantation (clinical psychologists and other specialists) to capture the key psychosocial and experiential aspects of the transplant process. A semi-structured moderating guide was used by the facilitator, and the questions are listed in **Table 1**. Each session began with the establishment of group norms (confidentiality/privacy, turn-taking, and non-judgmental communication), followed by an initial segment of open-ended questions regarding aspects of healthcare—related to both patients and parents—that participants would have altered or improved throughout the transplantation process. In the second part of the session, targeted questions were asked concerning biomedical, psychological, and social factors that could have been improved but had not emerged spontaneously earlier.

**Table 1:** Focus group topics and key questions.

Phase	Adolescent focus group
Free open-ended questions phase	-What aspects would you improve during your visits to the hospital or during your admission to the hospital for the organ transplant you received? -Have you missed any kind of support or specific help related to the transplant from any kind of professional working at the hospital?
Directed open-ended questions phase*	-In terms of the care you have been given by the doctors and nurses, what (other) aspects would you improve during your visits to the hospital or during your admission to the hospital for the organ transplant you received? -In terms of psychological and emotional issues, what (other) aspects would you improve during your hospital visits or during your admission to the hospital for the organ transplant you received? -In terms of social support, what (other) aspects would you improve during your visits to the hospital or during your admission to the hospital for the organ transplant you received?

	Parent focus group
Free open-ended questions phase	-What aspects of your child’s health care during the organ transplantation process that he/she has undergone, either during the follow-up in outpatient appointments or during hospitalisation, would you improve? -What kind of interventions or clinical care have you missed during this process for your child? -What kind of interventions or clinical care have you missed during this whole process for you? -What aspects of health care would you improve for you as a parent of a child who has undergone an organ transplant?
Directed open-ended questions phase*	-On a medical level, what (other) aspects would you improve in the care of your child and you as parents during the solid-organ transplantation process? -On a mental health level, what (other) aspects of care for your child and for you as parents during the solid-organ transplantation process would you improve? -On a social level, what (other) aspects of the care for your child and for you as parents during the solid-organ transplantation process would you improve?

*Directed questions were asked only when participants did not spontaneously address specific aspects of one of the three areas of the biopsychosocial model.

The sessions were conducted via video call, audio-recorded, and automatically transcribed, respecting the confidentiality of the information at all times. The sessions were conducted by a member of the research team and not by the PI of this project (EFJ) to avoid bias (the PI knows most patients and parents). The facilitator was a female psychologist with a Master’s degree who had no prior relationship or familiarity with the participants. She was presented solely as a researcher to minimise bias toward mental health themes at the expense of biomedical or social dimensions. Moreover, she did not contribute to any subsequent phases of the study, including transcription, data analysis, interpretation, manuscript preparation or review.

Consequently, the automatically generated transcriptions were reviewed to 1) change the names of the participants using a code and 2) correct any transcription mistakes. The audio recordings were retained until the accuracy of the transcription process was confirmed. Transcripts were not returned to the participants for their comments or corrections. When needed, in anticipation of collecting more relevant information, participants were offered the possibility of conducting individual in-depth interviews after the focus group. The interviews were conducted using the same standards as mentioned above.

The study protocol was registered at clinicaltrials.gov on July 1, 2022 (NCT05441436) (17). Recruitment for this study began on October 21, 2022, and ended on February 8, 2024.

### Ethical considerations

This study was conducted in accordance with the principles established in the Helsinki Declaration, strictly respecting confidentiality and the requirements of Spanish (14/2007, 3 July 2007 on Biomedical Research; and Organic Law 3/2018, 5 December 2018) and European data protection regulations. This study was approved by the Research Ethics Committee of La Paz University Hospital (Madrid, Spain) (institutional code: PI-5223). Parents or legal guardians of patients (or, when applicable, patients of legal age) provided their written informed consent, for minor patients and for themselves, to participate in this study.

### Data analysis

Two coders (JGB and EFJ) independently analysed the data following the steps of the qualitative framework analysis (14). Records of coding, matrices, and analytical decisions were documented in an Excel file. Independent coding was used to enhance reflexivity and analytic rigour while minimising the potential influence of the principal investigator’s prior clinical familiarity with some participants. This was followed by ongoing consensus discussions between coders. Any coding discrepancies were reviewed in consultation with a third researcher experienced in framework analysis (RV) until agreement was reached.

The first step was familiarisation, which involved reading all the transcripts thoroughly, listening to the tapes, and listing key ideas/recurrent themes to identify a thematic framework (data immersion). A coding frame was developed based on these key themes. This involved entering brief summaries into the coding frame along with the page numbers of the original data excerpts, facilitating easy retrieval for further analysis.

Subsequently, a biopsychosocial model was systematically applied to all the data (indexing), and the data were rearranged according to the relevant sections of the thematic framework (charting). Data were structured hierarchically according to the biopsychosocial framework, with three analytic levels: domains (theoretical dimensions), themes (patterned responses emerging across participants within each domain), and codes/subcodes (specific meaning units derived from the raw data). This hierarchical mapping facilitated the identification of cross-cutting patterns between adolescents and parents. Charting was performed using columns (participants) and rows (domains/themes/codes/subcodes). The code allocation process used in this study was as follows:

-Any code relating to the physical body, medical treatment, symptoms, or medication was allocated to the *“Biomedical Domains”.*
-Any code relating to internal thoughts, emotions, mental states, or Mental health support was allocated to the *“Psychological and Emotional Domains”.*
-Any code relating to external systems (healthcare or educational), interpersonal relationships, institutional and bureaucratic challenges, or logistical and caregiving burden was allocated to the *“Social Domains”.*

Finally, the themes were refined and clearly defined. The researchers then compared the participants’ accounts to identify similarities, differences, and recurring patterns, and interpreted how these findings related to the study aims (14).

## Results

In this section, we present the participants’ experiences regarding solid-organ transplantation and biopsychological factors, resulting from the analysis of the transcripts of two separate focus groups (adolescents and parents) and one individual in-depth interview with an adolescent transplant recipient.

The patient group consisted of adolescents aged 12–17 years at the time of transplantation (mean age = 14.6 years; *SD* = 2.30), with a mean post-transplantation period of 13.6 months (*SD* = 6.50; range: 6–24 months). The second group consisted of parents of paediatric transplant recipients at age of 7 and 13 years at the time of transplantation (mean = 10; *SD* = 2.55 years) who had undergone transplantation, on average, 14.8 months ago (*SD* = 5.54; range: 10 to 24 months). The characteristics of the participants are presented in **Table 2**.

**Table 2:** Characteristics of study participants (*n* = 10).

Adolescent group	Sex	Autonomous community	Age at transplantation	Age during participation in focus group	Time since transplantation to focus group	Type of transplant received
Adolescent 1	Female	Madrid	14	15	6 months	Kidney (deceased donor)
Adolescent 2	Female	Madrid	13	15	13 months	Heart
Adolescent 3	Female	Madrid	17	18	12 months	Kidney (deceased donor)
Adolescent 4	Male	Madrid	12	15	24 months	Lung
Adolescent 5	Male	Other*	17	18	13 months	Liver

Parent group	Sex of the parent and sex of the child respectively	Home region	Child’s age at transplantation	Child’s age during participation in focus group	Time since their child’s transplantation	Type of transplant their child received
Parent 1	Female/Female	Other	8	9	10 months	Heart
Parent 2	Male/Female	Madrid	13	14	14 months	Heart
Parent 3	Female/Male	Other	7	8	11 months	Kidney (living donor)
Parent 4	Male/Male	Madrid	10	12	15 months	Hepatorenal
Parent 5	Female/Male	Madrid	12	15	24 months	Lung

*Other autonomous communities are not specified to preserve participant confidentiality, as this level of geographic detail could increase the risk of identification in a small and specialised sample.

In the adolescent group, four codes emerged regarding the biomedical domains (encompassing the theme “*Physical Experience of Illness & Circumstances related to its Treatment*”). Two codes emerged regarding the psychological and emotional domains (theme titled “*Emotional Well-being & Mental Health Support”*). In turn, the social domains generated six codes, of which four codes and three subcodes were grouped under the theme “*Logistics & Healthcare System Interaction*” and two codes covered the theme “*Social & Academic Impact*”.

In the parent group, one code and three subcodes were generated regarding biomedical domains (theme “*Quality & Experience of Clinical Care related to Physical Illness*”). Three codes and two subcodes covered psychological and emotional domains (grouped under the themes “*Cognitive & Emotional States*” and “*Family Mental Health Needs*”). Finally, eight codes were derived from social domains covering the following three themes: “*Logistical & Caregiving Burden*”, “*Institutional & Bureaucratic Challenges*” and “*Social Support & Advocacy Networks*”.

Across both groups, participants identified all biopsychosocial domains as important aspects of the transplant experience, although social factors emerged as particularly salient ones. Differences between adolescents and parents were evident in the emphasis placed on specific domains and topics, as reflected in the coding of each group. For example, academic concerns emerged as a prominent theme among adolescents but were only minimally discussed by the parents. A selection of illustrative quotations is presented below, organised according to the three overarching themes—biological, psychological, and social—to provide a brief, representative overview of participants’ perspectives.

### Adolescent group:

- Biomedical domain: *“Before the transplant, I was diagnosed with renal failure from one day to the next, and I had no symptoms or anything... So, I was very shocked by that.”*
- Psychological domain: *“I wish the transplant had gone better... I had a lot of panic attacks in the ICU because of my rejection.”*
- Social domain: *“Truth be told, I had a really tough time because I missed a lot more school than usual (four and a half months), and I was really stressed out because I was at risk of repeating a year, which isn't ideal during high school...”.*

### Parent group:

- Biomedical domain: *“[ … ] when our son was undergoing the operation, the surgeons came out to explain what was happening. You are waiting there for many hours, and the fact that they came out to provide us with updates … It seemed to us that we always received information”.*
- Psychological domain: *“There were good days and bad days, but I have been very nervous and upset. I did not know what was going to happen, and everything could change in five minutes (…). The worst time for me was the month after the transplant when my daughter was admitted to the ICU. I only noticed the shortcomings of nurses, assistants, and doctors. I just wanted everyone to be with my daughter. I did not want to accept that it was my daughter’s body that was sick; instead, I tended to blame others. Now that I am out of that rollercoaster, I look back and can understand that it was very difficult, very complicated, and I can see that everyone did their best. I am very grateful now and I have come to realise it was my nerves, the tension, the fear…”.*
- Social domain: *“There is significant stress for us as parents trying to be in two places at once – work and the hospital. The challenge is even harder if you have more children. We need to make a living but it’s hard to balance the fact that you have to dedicate yourself to your son who is in post-operative care, at the same time that you are leaving work and family behind.”*

More exact quotations of the participants’ contributions during the focus groups and the individual in-depth interview can be found in the **Supplementary Material** (**Appendix 1**).

No further focus groups with additional participants were required, as theoretical sufficiency and a high degree of theme recurrence had been achieved. **Table 3** presents an overview of the themes and codes/sub-codes that emerged during the focus group sessions.

**Table 3:** Domains, themes, and codes addressed by each focus group: Adolescents and parents.

Adolescent focus group		
Themes		
*Biomedical Domains*	*Physical Experience of Illness & Circumstances related to its Treatment*	
*Codes*	Perception of long hospital admission	
	Impact of clinical diagnosis	
	Access to medication	
	Symptoms	
*Psychological and Emotional Domains*	*Emotional Well-being & Mental Health Support*	
*Codes*	Anxiety	
	Mental health care	
*Social Domains*	*Logistics & Healthcare System Interaction*	
*Codes/subcodes*	Distance to the hospital	
	Hospital Staff	*Satisfaction with humanised interaction*
		*Dissatisfaction with interaction*
	Hospitalisation ward	
	Outpatient care	*Waiting times*
*Social & academic impact*		
*Codes*	Limited social interactions	
	Academic challenges	

Parent focus group		
*Biomedical Domains*	*Quality & Experience of Clinical Care related to Physical Illness*	
*Codes/subcodes*	Medical care/attention	*Satisfaction with clinical care*
		*Excessive clinical care*
		*Experience being a donor*
*Psychological and Emotional Domains*	*Cognitive & Emotional States*	
*Codes*	Attitudes, beliefs and expectations	
	Wishes and desires	
*Family mental health needs*		
*Codes/subcodes*	Mental health care	*For the child*
		*For other family members*
*Social Domains*	*Logistical & Caregiving Burden*	
*Codes/subcodes*	Distance to hospital	
	Caregiver leave at the workplace	
	Outpatient care	*Waiting times*
	Balancing/conciliating hospital visits with work and family responsibilities	
*Institutional & bureaucratic challenges*		
*Codes*	Bureaucratic challenges	
	Laws, social aids, and information received regarding them	
*Social support & advocacy networks*		
*Codes*	Need of creating a peer/family support network	
	Advocacy for transplanted children to be recognised as a collective	

## Discussion

To the best of our knowledge, this is one of the few qualitative studies to examine perceived unmet needs throughout the paediatric organ transplantation process, comparing the perspectives of adolescent transplant recipients and parents of paediatric transplant recipients across biopsychosocial domains, and involving multiple types of solid-organ transplants.

We intentionally included adolescents who had undergone transplantation between six months and two years prior to participation. This timeframe was selected to include both those with relatively recent and emotionally intense experiences and those who had progressed further in recovery, allowing for greater perspective and reinterpretation of their experiences. Additionally, this range enabled participants to benefit from the shared insights of peers or parents who had lived with the transplant for a longer period. The findings from both focus groups are discussed, with comparisons made to the existing literature, followed by a comparative analysis of the topics that emerged in each group.

A key finding was the mismatch between parents’ and adolescents’ concerns about academics—parents rarely mentioned academics, whereas adolescents often reported intense anxiety about their academic performance. This difference may be understood in light of developmental factors, as adolescents who undergo transplantation during this stage of life often place significant importance on keeping pace with their peers and may struggle with setbacks, particularly as they already experience that a significant portion of their time has been devoted to hospital care (18). In contrast, parents typically have a broader and more distant perspective, which allows them to recognise that their child has already faced considerable challenges and that academic progress can reasonably proceed at a slower pace. Of course, these patterns do not necessarily apply to every adolescent or parent, but they highlight the value of considering both perspectives when designing ongoing support and prehabilitation interventions. This anxiety was heightened by the perceived lack of educational support and adaptations. Also, existing research indicates that adolescent transplant recipients may experience cognitive and neuropsychological impairments (19, 20), which may contribute to the aforementioned perceived problems. This underscores the need for more robust academic support systems tailored to adolescents’ unique circumstances (21).

Conversely, key issues that emerged for both informants were inconveniences regarding the distance to the hospital, long waiting times for outpatient hospital visits, the need for peer support, satisfaction with the humanised care provided by healthcare workers, and the relevance of mental health support during the transplant process. In relation to the latter, all participants received follow-up care from the Mental Health Consultation-Liaison team, composed of clinical psychologists and/or psychiatrists, throughout the pre- and post-transplant phases. None of the adolescents or parents had a prior history of psychiatric diagnosis beyond the expected emotional reactions associated with the transplant process. Even so, participants consistently expressed appreciation for having access to specialised mental health professionals, and several highlighted the importance of being able to address thoughts of death and the psychological impact associated with transplantation.

In addition, one participant described further distress linked to immigration-related challenges, illustrating how transplant-related stressors intersect with broader contextual vulnerabilities. These findings reinforce the need for a holistic approach to psychosocial care, and align with emerging research that is increasingly examining the mental health profiles of paediatric transplant populations in order to improve the integration of psychological support into routine care (2, 8, 17, 22).

When considering mental health outcomes, the potential impact of pharmacological factors should also be acknowledged. Corticosteroids and other immunosuppressive agents may induce visible side effects, such as acne, weight gain, and striae, as well as mood alterations that can influence adolescents’ body image, social interactions, and overall psychosocial adjustment. Notably, these effects were not spontaneously raised by participants during the focus group discussions, suggesting that they were not a prominent feature of participants’ narratives within this study. From a clinical perspective, this highlights the importance of proactively exploring medication-related physical and emotional side effects during follow-up care, as these may remain unspoken unless directly elicited. Systematic enquiry into these domains may support a more comprehensive understanding of adolescents’ psychosocial adjustment and care needs over time.

Revisiting the concerns shared by both adolescents and parents, one recurring issue was hospital accessibility. For example, Adolescent 5’s experience illustrates a common challenge faced by patients who must travel from other regions to undergo transplant procedures. As Spain’s national reference centre for all paediatric transplants, La Paz University Hospital receives patients nationwide. Long-distance travel, often by ambulance or plane, adds logistical and emotional strain. Families face financial burdens from accommodation and related expenses, and separation from loved ones during critical times adds emotional distress. These experiences are consistent with previous research showing that parents of children with chronic illnesses frequently report elevated levels of anxiety and stress related to managing treatment logistics and family life (9).

Peretz et al. (23) investigated the psychological and financial challenges associated with long-distance travel for liver transplantation. Although patients typically maintained confidence in their healthcare providers and did not perceive their care as compromised by distance, many caregivers—especially women—reported frustration with limited visitation opportunities. Moreover, the study stated that the financial strain of travel contributed to 70% of patients expressing a preference for undergoing the procedure locally. Our findings, along with previous literature, suggest that it is important to consider more decentralised transplant services or enhanced support for travel and accommodation to alleviate the burden on patients and their families. However, this should be interpreted cautiously given the exploratory nature of this qualitative study and, in some cases, its basis in individual participant perspectives.

Most of the participating parents were mothers. This pattern reflects what has been consistently documented in the paediatric chronic illness literature, where mothers tend to assume the primary caregiving role and report higher levels of caregiving stress than fathers (5). Although our study did not include a gender-based analysis of caregiver experience, acknowledging this is important, as the emotional, social, and occupational burden of transplantation may be differently distributed across mothers and fathers. Future research should explore how gender shapes caregiving demands, access to support, and psychosocial impact, and whether interventions need to be tailored to these factors.

In addition, extended outpatient waiting times were generally poorly tolerated, and prolonged hospital stays were particularly challenging for adolescents. This highlights an area that requires urgent improvement to enhance patient and caregiver satisfaction, as previously noted in the literature on paediatric outpatient care (24). In response to these concerns, several practical measures could help reduce the burden of repeated visits and long hospital stays for patients. Coordinating appointments across specialties so that consultations and tests take place on the same day, when possible, would prevent families from having to return multiple times in a short period. Some routine follow-up activities can also be handled through online appointments, reducing the need for in-person attendance when clinically appropriate. In addition, assigning a single care coordinator to assist with scheduling and communication may help families navigate the process more efficiently and effectively. Finally, offering structured support during prolonged hospitalisations, such as accommodation options and school-continuity resources, could ease both the logistical and emotional strain described by participants (25).

Furthermore, both parents and adolescents identified social issues as their predominant concerns over biomedical and psychological issues. This finding is consistent with current research, which shows that patients and families face enduring psychosocial risks long after the initial post-transplant phase (26). Consequently, it is essential to make social support a central component of interventions to effectively address the well-being of patients and their families. Among the topics that emerged at the social level, participants highlighted the value they would have found in peer and family support groups. Adolescents, in particular, reported being significantly affected by reduced social interaction during hospitalisation and with isolation protocols. Such isolation can be distressing, underscoring the need for effective communication channels and support systems to protect patients’ mental health (27). This is consistent with the study by Anthony et al. (28), who reported high satisfaction among participants in an online peer mentorship program for paediatric transplant patients. These support groups offer essential emotional connections and community, enhancing coping strategies for both patients and their families.

In addition, one of the notable social-level suggestions highlighted by participants in the parent group was the potential benefit of having a unified reference figure from social work to assist with bureaucratic procedures systematically rather than reactively. This would alleviate the administrative burden on families, allowing them to focus on the emotional and psychological aspects of care. There was a strong desire for a dedicated reference person who could provide expert guidance on complex laws and regulations related to medical care, economic aid, and work-leave entitlements. For instance, in Spain, the Compensation for Leave to Care for Children with Cancer or Serious Illnesses (CUME, acronym in Spanish) (29) allows working parents to take leave to care for children undergoing serious medical treatment, with financial support provided in their absence. However, one parent noted that they only learned about this social aid during the focus group discussion of this project and wished that they had been informed earlier.

Balancing family responsibilities with work commitments emerged as a significant challenge for parents, consistent with the existing literature that categorises their difficulties into three domains: work, family, and personal. Common workplace issues include limited access to flexible arrangements, absenteeism, and unsupportive employer attitudes. Family-related challenges involve securing childcare, managing time, and meeting both the needs of an ill child and broader family obligations. On a personal level, parents frequently experience stress, uncertainty about the future, and the feeling of *“having to do it all”* (30). This calls for more flexible support systems to assist families in effectively managing their dual responsibilities (6).

Additionally, addressing the needs of siblings was another critical point. The existing literature indicates that siblings of children with chronic conditions face unique challenges that affect their health-related quality of life, including negative emotions, separation anxiety, increased responsibilities, and feelings of exclusion (31). Feelings of abandonment among siblings must be mitigated by including them in the entire care process and offering psychological support when necessary. This holistic approach ensures that the emotional needs of the family are met, thereby promoting a more cohesive support system.

It is possible that some of these perceived needs could be partially addressed by the suggestions made by one parent during the focus group discussion. They proposed that children undergoing transplantation should be legally recognised as a distinct group for disability-related considerations. Such recognition could streamline access to the necessary support services for these children and ensure that parents receive accurate and timely information about the condition. Once more, these suggestions should be interpreted with caution, as they were derived from a small exploratory qualitative study. To further synthesise the needs identified in this study and outline potential strategies to address them, consistent with the main aim of this work, **Table 4** presents a summary of prehabilitation and ongoing support recommendations throughout the transplant process for paediatric solid-organ recipients.

**Table 4:** Prehabilitation and ongoing support recommendations throughout the transplant process in paediatric solid-organ recipients.

Domain	Recommendation	How it can be offered	Basis from focus groups
Preparation & expectation management	Educate patients and families on what to expect before, during, and after transplant	Pre-transplant workshops, informational sessions, guided hospital tours	Adolescents and parents expressed uncertainty about recovery and hospital procedures
Psychological support	Provide early and continuous mental health support before, during, and after transplant	Individual or family counselling, online mental health follow-ups, integrating psychological screening into routine care	Adolescents and parents emphasized the importance of managing anxiety, stress, and fears about death, highlighting the value of ongoing support
Academic support	Support school reintegration and individualized learning needs	Liaise with schools, provide tutoring, flexible assignments, and individualized educational plans	Adolescents reported anxiety about academic performance and insufficient adaptations, showing a need for structured academic support
Peer & family support	Facilitate peer connections and family support, including siblings	Online or in-person peer groups, workshops for families, sibling-specific interventions	Isolation and limited social interaction were major stressors; siblings also faced emotional and practical challenges
Hospital access & logistics	Minimize travel burden and logistical challenges	Help coordinate transportation, provide accommodation near the hospital, reimburse travel costs when possible, offer online follow-up	Long-distance travel and separation from family caused emotional and financial stress for families
Medication side effects	Monitor and manage effects of immunosuppressive drugs	Regular check-ins about physical and emotional side effects, patient education, early intervention for mood or body image concerns	Adolescents prioritized transplant recovery, but side effects like weight gain, acne, or mood changes can affect adjustment
Social work support	Provide a dedicated reference for navigating administrative, financial, and care-related processes	Social worker to coordinate financial aid, travel and lodging support, medical leave entitlements, and access to services	Parents highlighted the burden of bureaucracy and the need for proactive, coordinated guidance
Patient-centred communication	Ensure clear and compassionate communication	Staff training in empathetic communication, structured information sessions, written or digital materials	Both adolescents and parents valued humanized interaction and clear explanations about the care process

Finally, humanised interaction by healthcare personnel was highly valued by both patients and parents, underscoring the importance of empathy and compassion in medical care (32).

### Strengths and limitations

This study has some limitations. It included only two separate focus groups from a single hospital. Nevertheless, these participants were purposively selected based on information power criteria (16), thus ensuring a diverse view of the potential circumstances surrounding the paediatric transplant process in accordance with sociodemographic and clinical variables (organ type, sex, and socioeconomic status). In accordance with the foundational principles of qualitative methodology (33), this study prioritised theoretical sufficiency over statistical power, as the sample size is less critical than in quantitative research. Empirical evidence supports that theoretical sufficiency can often be reached with a limited number of focus groups or interviews, particularly when investigating homogenous populations with well-defined research objectives (34). Furthermore, smaller yet rigorously conducted focus groups may generate richer and more nuanced insights than larger and less targeted discussions (35).

Another limitation was the heterogeneity of the parent group, which included parents of three children under 12 years of age, whereas the adolescent group consisted solely of participants aged 12–17 years at the time of transplantation. This approach was necessary because of limited access to the paediatric solid-organ transplant population and the difficulty of recruiting participants who met multiple important criteria, including representation of different organ types, willingness to actively participate in focus group discussions, and inclusion of unique cases such as an immigrant single mother and a parent who had served as a donor. While this strategy allowed for a more comprehensive exploration of parental experiences, it may have introduced variability in perspectives within the parent group and should be considered when interpreting these findings. Future studies with larger sample sizes may enable separate analyses by age group, donor status, and other relevant characteristics.

Furthermore, another limitation of the study is that conducting focus groups online may have resulted in the loss of some non-verbal information. However, this approach was chosen to facilitate the participation of participants from geographically diverse regions, who had returned to their home cities, avoiding the need for long-distance travel. Limiting recruitment to Madrid would not have provided a sufficient sample or captured the diverse experiences of transplant recipients, particularly those related to the burden of receiving medical care away from their homes.

In addition, the study may have been influenced by the structure of the interview guide, which included prompts specifically focused on aspects that participants would have liked to improve. Consequently, the data collection process has been oriented toward identifying deficits and unmet care needs rather than capturing the full breadth of participants’ transplant experiences. While this focus is consistent with the aims of the study, it should be acknowledged that it may have shaped the type and emphasis of the information generated during the focus groups.

Furthermore, participants were recruited with the expectation that they would actively engage in focus group discussions, which may have introduced a degree of selection bias toward more motivated or communicative individuals. The group format may also have been influenced by social desirability effects, potentially shaping the openness of some contributions.

To minimise bias, the principal investigator did not conduct the focus groups. However, he did have prior clinical relationships with some of the participants, which may still have influenced participants’ responses despite these precautions. Moreover, transcripts were not returned to participants for verification, thereby limiting the possibility of member checking. Furthermore, some participants had received mental health follow-up care, which may have influenced their perceptions and potentially contributed to a more favourable appraisal of psychological support services.

Finally, dedicated qualitative software, such as NVivo or ATLAS.ti, was not used in this study. Instead, data were managed and coded using structured spreadsheets designed specifically for framework analysis, which allowed us to systematically map the themes within the biopsychosocial model across participants and domains. Given the modest dataset and structured nature of the analytical framework, manual organisation through spreadsheets was considered sufficient and appropriate for this study. This approach aligns with established qualitative research practices, particularly when the analytical framework and data volume allow for direct comparison and indexing without additional software (14).

Ultimately, it is important to note that despite the potential for conformity among participants inherent to the focus group methodology, both groups in this study demonstrated a range of divergent perspectives and complex opinions, reflecting the participants’ willingness to express disagreement and articulate differing viewpoints.

## Conclusions

In conclusion, this study highlights several important areas for improving the care and support of adolescent transplant recipients and the families of paediatric transplant recipients. These include enhancing academic support, improving interactions with healthcare professionals, and strengthening access to mental health care, alongside broader family and peer support, including attention to siblings. Flexible support arrangements that help families balance caregiving responsibilities with work commitments are also needed, as this remains a common challenge. Extended hospital stays and travel-related demands were poorly tolerated, underscoring the importance of better coordination of care and more practical support for families who must travel long distances, including consideration of accommodation needs to reduce logistical and financial strain. In addition, the involvement of a dedicated social work professional to support families with administrative and bureaucratic processes may help address some of the complex needs identified.

Collectively, these findings may inform the development of future multifaceted prehabilitation and ongoing support interventions, as well as clinical recommendations, across the paediatric solid-organ transplantation pathway.

## Data Availability

The original contributions presented in the study are included in the article/**Supplementary Material**. Further inquiries can be directed to the corresponding author.
