# Supplementary material for: Experiences of parents and adolescents throughout the solid-organ transplantation process: a focus group study
Source: Front Psychiatry. 2026 Aug 5;17:1633641. doi: 10.3389/fpsyt.2026.1633641 (PMC13488533; doi:10.3389/fpsyt.2026.1633641)
Supplement: Supplementary file 1 [file Table1.docx]

**APPENDIX 1 – SUPPLEMENTARY FILE**

**ADOLESCENT FOCUS GROUP**

Biomedical Factors

**Perception of long hospital admissions.** Most patients felt that their hospitalisation was excessively prolonged. One participant mentioned that it became particularly burdensome once she experienced clinical improvement.

*“I felt it was overwhelming to be hospitalised for a very long time”.*

*“When I started to get better, I got tired of being there [hospital ward] because I was there for a long time”.*

**Impact of clinical diagnosis.** One adolescent requiring a kidney transplant described the impact of suddenly and unexpectedly receiving a diagnosis of a chronic renal condition.

“*Before the transplant, I was diagnosed with renal failure from one day to the next, and I had no symptoms or anything... So, I was very shocked by that.”*

**Access to medication.** Certain participants mentioned that access to medication could be problematic, as it was intermittently unavailable at local pharmacies.

*“Sometimes health professionals had trouble getting me certain medications because they were either out of stock or temporarily discontinued”.*

**Symptoms.** The most common symptoms were fatigue, sickness, and concentration difficulties.

*"I felt very weak and tired at the hospital and at home, I didn't want to get out of bed… I felt a lot of stress".*

*“My immune system was very low and it made it [studying] more difficult (…). I could not concentrate, grasp the information well, understand what the teachers were saying…”.*

Psychological and Emotional Factors

**Anxiety.** Most participants reported anxiety as their predominant emotional state. One adolescent who became a kidney recipient reported frequent panic attacks following renal graft rejection.

*“To be honest, I experienced a lot of anxiety after the transplant, especially at the hospital”.*

*“I wish the transplant had gone better... I had a lot of panic attacks in the ICU because of my rejection.”*

**Mental health care.** Adolescents rated contact with a mental health professional (clinical psychologist/psychiatrist) as a positive intervention. One adolescent who received a lung transplant noted that he benefited from psychological assistance to cope with medical tests that caused him anticipatory anxiety.

*“I value psychologists the most. Before my transplant, I was anxious and stressed. The psychologists helped calm my nerves during that time, because just thinking about the transplant procedure made me anxious.”*

Another adolescent who underwent a liver transplant commented: *“The psychologists had to intervene because I was clearly reluctant to undergo the lumbar puncture”.*

Social Factors

**Distance to hospital.** Adolescents who had to travel between cities for medical attention reported some inconveniences, such as transportation and being far from their loved ones.

*“Transport to medical appointments, that's the only problem (...) the type of vehicle [ambulance] was uncomfortable …”.*

*“Personally, I strongly would have preferred being in my homeland [region of Spain], I missed having friends visit [during hospital admission] because I was in Madrid.”*

**Hospital Staff. Satisfaction with humanised interaction.** Overall, there was widespread satisfaction with the interpersonal care provided by the hospital staff among the participants, which significantly eased their hospital visits and admissions.

*“All the efforts made by the nurses, doctors, and orderlies to create a positive experience for us despite the challenges (...). Their efforts ultimately gave me hope for life after the transplant and, at the same time, made my time there a little brighter.”*

Only one adolescent held a more critical view of this matter.

*“I think I would improve the interaction from the nurses or assistants (...). Sometimes, they would come in with a bit of a bad mood or something like that. That's the only thing I would change.”*

**Hospitalisation ward.** One adolescent who was a kidney recipient expressed discontent with the formal aspects of the hospitalisation ward, particularly the distribution of patients in the rooms.

*“In other surgeries, such as the catheter dialysis (...) I was in a shared room with a two-month-old baby. Therefore, the truth is that I had a very, very bad time (...). I told my doctors “I want to leave now because I cannot stay here any longer”. Because imagine, a two-month-old baby girl who would not stop crying all night long.”*

**Outpatient care.** Adolescents complained about their waiting times.

*“I had to wait for two or three hours for some appointments (…). When I had to go there every three months, I was like – I do not want to go because I am going to be there for hours....”*

**Limited social interactions.** All the adolescents agreed that this was one of their biggest challenges.

*“When I was on dialysis, I had very limited freedom. Travelling, for instance, was quite challenging because of the machine and logistical issues involved. It restricted me during a time when my friends were enjoying more independence and going out at 16-17 years old. This was particularly tough for me.”*

**Academic Challenges.** All adolescents, particularly those in their final years of high school, expressed the negative impact of transplantation on their academic performance.

*“Truth be told, I had a really tough time because I missed a lot more school than usual (four and a half months), and I was really stressed out because I was at risk of repeating a year, which isn't ideal during high school...”.*

*"When you return to high school after a transplant, it can be in the middle of the academic year… it can be disorienting because your classmates are further ahead than you".*

Many expressed frustration and despair over losing valuable school time due to long waiting hours for outpatient hospital appointments.

*“The truth is that I was in my first year of high school and waiting two or three hours for medical check-ups wasted a lot of my time”.*

One adolescent expressed dissatisfaction with the lack of curricular adaptation.

*"When I arrived, I had missed most of the first term, and unfortunately, the teachers did not offer much assistance. They immediately gave me exams without allowing me time to catch up, which caused me to struggle. Now, I find it challenging to keep up with the others.”*

Finally, some adolescents expressed limitations in receiving educational support either at home or in the hospital, indicating that their assistance was insufficient. One adolescent, who was entitled to the Home Education Support Service, reported not benefiting from this service at all.

*“They told me that since I was in my final school year, I could request home education for when I had to stay home in isolation, to help me keep up and not fall behind. However, the teacher never showed up (…). It really frustrated me, as it has been very noticeable, especially in mathematics which is a subject where a lot of progress was made”.*

*“When I was in the hospital, there were two teachers who came to help (…), but I could see that they didn’t have enough time because there were too many children, too many people…”.*

**PARENT FOCUS GROUP**

Biomedical Factors

**Medical care/attention.** Overall, most parents expressed a high level of satisfaction and gratitude for their children’s medical follow-up and care throughout the transplantation process.

*“We are very happy and grateful with the medical attention our child received. The truth is that the doctors were always excellent.”*

*“It seemed very human to us that when our son was undergoing the operation, the surgeons came out to explain what was happening. You are waiting there for many hours, and the fact that they came out to provide us with updates... It seemed to us that we always received information; they always asked you how you were doing and so on....”*

However, one parent reported a subjective experience in which medical attention seemed excessive on some occasions.

*"There are times when you get overwhelmed by all the medical attention. When you think about it rationally, you realise that it is good that they are so thorough in attending to him and monitoring his condition. They want to ensure that everything is under control and that they are doing well. It's just that we spend all day at the hospital, and at first glance, it can feel excessive.”*

One woman expressed dissatisfaction with the medical attention she received after donating her kidney to her son.

*"In my particular case, I was the donor, and naturally, all the attention was focused on the child, not the parent. However, I had also undergone surgery… In some way, I felt neglected, receiving minimal attention as an adult. This lack of care was concerning, setting aside a more emotional aspect, I could have experienced a medical complication.”*

Psychological and Emotional Factors

**Experiences, attitudes, beliefs and expectations.** Parents shared their experiences and thoughts regarding their children’s transplantation process.

*“There were good days and bad days, but I have been very nervous and upset. I did not know what was going to happen, and everything could change in five minutes (…). The worst time for me was the month after the transplant when my daughter was admitted to the ICU. I only noticed the shortcomings of nurses, assistants, and doctors. I just wanted everyone to be with my daughter. I did not want to accept that it was my daughter's body that was sick; instead, I tended to blame others. Now that I am out of that rollercoaster, I look back and can understand that it was very difficult, very complicated, and I can see that everyone did their best. I am very grateful now and I have come to realise it was my nerves, the tension, the fear….”*

*“I felt a lot of fear; I wanted someone to tell me exactly what a heart transplant was like, what could happen… As time went on and I met more families, I came to understand that the anxiety I had about knowing what would happen could not be eased by another parent. I realised that my daughter's transplant would be unique to her. They might have performed fifty transplants before, but I understood that when they transplanted her, she would face her own set of complications, just as other children would face theirs.”*

**Wishes and desires.** We asked parents if there was anything they wished they had had or felt they had missed during their child's transplant process... One parent expressed that they missed the opportunity to convey gratitude to their son's donor.

*"There is one part that has always stayed with me, like a thorn in my side. I think it may be dictated by protocols that it cannot be done, but I always feel a debt of gratitude to the donors. It is like you receive the donation, and have not been able to thank them for anything. It is something that makes me feel like I have a debt I cannot repay. I imagine that it probably cannot be done, and so on. But emotionally, if there is one thing I still need to do, it is to thank the donor."*

**Mental health care.** Parents discussed which stages of the transplant process they believed psychological support was most useful or necessary for their children. Several parents noted that their children began expressing sudden concerns about death in the period leading up to the transplantation.

*“When the idea of the transplant was introduced, she did need psychological treatment. The treatment was not very, very demanding, but we did notice that from then on, she needed it, as she had changed a little bit. She started to be quieter, she started to think more... Anyway, she started asking us things about death... Now, we still go to the psychologist, but she is doing very well.”*

*"In the months before the transplant, I noticed my son was very worried about death and related issues. I do not know if it’s a normal concern or not at that age, but I think psychological care beforehand could have helped.”*

Another fundamental aspect discussed was the necessity of providing psychological support not only for the child undergoing transplantation but also for other family members, such as siblings, who also experience distress during this period and may feel overlooked.

*“My eldest daughter had a very bad time and kept quiet about it. I think siblings are also victims in a way, and we only realise this later when everything calms down. When the calm comes after the tsunami, then you start to notice the other little people around you, reminding you that they exist and that the situation has also affected them (…). Psychological care is provided to the parents, but in my case, the siblings are somewhat left in no man's land (…). They are left alone at home with their grandparents or other family members and are often overlooked as the days go by.”*

Social Factors

**Distance to the hospital.** Parents expressed the burden of not being able to receive medical attention in their own city, which forced them to commute to the city where the hospital is located. This disrupted their normal lives and resulted in significant economic losses.

*“Since we are from far away [a Spanish region far from the city where the hospital is located], we had to move and stay in Madrid for the whole time we were in the hospital and for the post-transplant period, as you have to go up to four times a week or several times a day to the hospital. These trips to Madrid have a cost and they do not allow you to make up with overtime at work.”*

*“If you live near [the hospital] that's fine, you have it easier, but those who do not live near have to spend a lot of money on travel, for example, on lodging… and that, in the end, of course, is a stress, because you say gee... Can I do it? Can I afford it? I think that's a big problem.”*

Finally, some expressed how difficult it was to be far from their loved ones and unable to leave the city while their daughter was on the heart transplant waitlist.

*“We were on the waitlist for three years. We have lived in Madrid for nine years since my daughter was born, but our family is in [a region in the south of Spain]. It was a tough three years because we could not leave Madrid.”*

**Need of creating a peer/family support network.** Parents agreed that it would have been beneficial for them and their children if the hospital had offered a peer/family support network, allowing them to meet other children and families in similar situations.

*“It would be good for the children, especially in a medical specialty such as lung transplantation, which is not very common as there are few cases and few children who have gone through this process and are alive; to be able to do some kind of activity for them to interact more, get to know each other, share their experiences. Because in my son's case, he is now entering adolescence, and just two weeks ago, he met another boy who had also received a lung transplant, and for him, it was something cool – he has gone through the same thing as me – he said. They need to share that information, they need to hear the opinion of someone else who went through the same process.”*

*“Those three years [waiting for her daughter's heart transplant] I would have liked to have met more parents in the same situation”.*

**Caregiver leave at the workplace.** One parent, although grateful for the flexibility offered at work, wished that they had known what the law states about time off in such situations.

*“At work, they told me to take as many days off as necessary, but I thought maybe in other places there are more limited days of time off, or there is an established protocol… Because especially after the transplant you have to be at the hospital for a long time and I think it is more than the legal time allowed".*

**Outpatient care.** Some parents complained about the waiting times.

*“I don't know what the hospital protocol is like or anything, but you arrive with a child*

*who has been very ill and still is, and you have to wait three hours for an appointment… I do not understand why they do not give them a card or something to help respect the appointment times. If they had to wait 40 minutes, okay, but what is not acceptable is three or four hours. And if you have another appointment that day, it means that for two consultations, you could be losing up to five or six hours.”*

Concerns were also voiced about remaining in a crowded waiting room with an ill child exposed to many other children for extended periods.

*“When you are in a waiting room with 40 other people and you have to stay there for three or four hours, you are putting your daughter at risk of catching anything. They are children who have been transplanted and well, my daughter was also weak before she was transplanted, so, I think that should be improved.”*

**Balancing/conciliating hospital visits with work and family responsibilities.** This aspect was a shared concern among the participants of the group.

*“There is significant stress for us as parents trying to be in two places at once – work and the hospital. The challenge is even harder if you have more children. We need to make a living but it’s hard to balance the fact that you have to dedicate yourself to your son who is in post-operative care, at the same time that you are leaving work and family behind.”*

*“In my case, we spent six months in the hospital, from the time she was transplanted until she came home. A long time passed and my other children felt in some way abandoned. – Where are my parents? – they must have thought. And of course, you get home, and you need to sleep because you are knackered, and all you really want to do is go back to the hospital because my only obsession was being in the hospital. My eldest daughter had a bad time because, of course, I could not look after her, my husband could not look after her...We spent most of our time at the hospital.”*

**Bureaucratic challenges.** There was widespread discontent with how time-consuming and inefficient bureaucracy can be, especially when one has a child who is ill and needs full attention.

*“Paperwork is very slow and time-consuming in general, and even more so for those of us who are parents of children who are ill and need all our time; they need us to be with them 100%. We do not have time to be doing all these procedures and going from one place to the other because the children need 100% of the parents' care.”*

*“You have to navigate through a lot of bureaucracy. For instance, with my daughter, they approved her dependency status because before her operation, she had only half a heart and needed oxygen. However, it took two and a half years to get that approval. Another frustrating issue is how they assess her disability level; it can change if her condition improves or after surgery, and then you are back to fighting bureaucracy again, all while caring for a sick child at home. It is unpredictable; today they grant you assistance, but tomorrow they might revoke it. For instance, after a successful transplant, she might need a tracheotomy, so the situation keeps changing, and you cannot constantly update the administration about the evolving circumstances.”*

**Laws, rights aid, and information received regarding them.** Some parents were disappointed to discover, very late or not at all, certain laws that could have provided crucial support during their child's transplant.

*“Nobody informed us that there was a law that covered parents with children with serious illnesses and allowed you to reduce your working hours from 50% up until 100% (…). I found out very late that I could have dedicated myself exclusively to the care of my son and still receive my full salary.”*

They also missed having a reference figure to whom they could direct their questions regarding laws and available social aid.

*“I missed having a specific figure to inform us about all the regulations, laws, aid, and procedures necessary when you have a child with special needs and requires special attention. There are likely many laws or regulations, whether at the state or community level, that I am unaware of and may only learn about in the future. I miss having a unified figure to guide us. Today, I feel we are excluded from all the help offered by the country and the community because we simply do not know about it. There is no one to inform us.”*

One woman described feeling misinformed and struggling to understand the processes because she was an immigrant and was unfamiliar with many laws and regulations.

*"I do not know the policies, I do not know the country’s system... I do not know how to get an appointment to be informed... It has been very hard for me. I have been doing this for three years now, and I do not see that there is an organisation offering help to a parent who does not know or is not from this country. I do not have the degree of Dependency…I have knocked on the doors of the social workers and I have not managed anything.”*

A woman expressed disappointment that the psychological support provided by national insurance is not available in her homeland for children who have undergone transplants.

*“Here in [other Spanish region], psychological support for a transplanted child is not considered necessary, so we had to pay for private consultations. Unlike getting a prescription for medication, psychological treatment takes time, and sometimes the financial burden is too much to bear. We were told by the national insurance that unless there is something like Attention-Deficit/Hyperactivity Disorder (ADHD), or a condition of the autistic spectrum or a dysfunctional family, that my son was not eligible for psychological therapy.”*

**Parents' advocacy for transplanted children to be recognised as a collective.**

*“My son faces both nephrological and visual challenges. The main issue is that transplanted children are not recognised as a distinct group. For instance, my son receives comprehensive support for his visual impairment from the Department of Education, including a social worker, teachers, and adapted materials, because individuals with visual impairments are recognised as a group. In contrast, transplanted children do not receive similar support; there are no counsellors or social workers available to us. For my son's visual impairment, we have regular visits, phone calls, appointments, and guidance on aids. However, for his transplant-related needs, there is minimal support and recognition. It is crucial for transplanted children to be acknowledged as a distinct collective group, similar to how other groups such as those with visual impairments or mobility issues are recognised and supported.”*
